# CURRENT AND FUTURE SERVICE USE, SATISFACTION, AND NEED FOR SERVICES AS DEFINED BY RURAL AND FRONTIER OLDER ADULTS

**DOI:** 10.1093/geroni/igad104.0517

**Published:** 2023-12-21

**Authors:** Nancy Karlin

**Affiliations:** University of Northern Colorado, Greeley, Colorado, United States

## Abstract

This study focused on differences for current service utilization and satisfaction, along with perceived future service use and need for rural versus frontier residing older adults. A sample of older adults (age 65+), stratified using United States government and State of Wyoming definitions for rural (n=70) and frontier (n=72) counties, were interviewed. Frontier respondents reported less education F (1, 141) = 10.19, p = .002, η2 = .068, monthly income F (1, 141) = 17.03, p = .001, η2 = .108 with fewer financial resources F (1, 141) = 10.32, p = .002, η2 = .069, experience with technology F (1, 140) = 4.53, p = .035, η2 = .032, and potential for telehealth F (1, 140) = 19.27, p < .001, η2 = .112, along with fewer computers F (1, 141) = 11.08, p < .001, η2 = .084, and less access to the internet F (1, 141) = 10.19, p = .001, η2 = .073. Differences were evident for recent use of senior nutrition programs and meal sites, dental care, and senior centers along with future use of senior nutrition programs and meal site location, food related services, hearing and vision clinics, and housing needs. Policy concerns have been identified for the funding of future senior housing, medical care, transportation, and food support in frontier and rural areas.

